# Pharmacological inhibition of key metabolic pathways attenuates *Leishmania* spp infection in macrophages

**DOI:** 10.1371/journal.pntd.0012763

**Published:** 2025-01-07

**Authors:** Elaine Carvalho de Oliveira, Rafael Tibúrcio, Gabriela Duarte, Amanda Lago, Léon de Melo, Sara Nunes, Gustavo Gastão Davanzo, Ana Júlia Martins, Bruno Vinagre Ribeiro, Deborah Mothé, Juliana B. P. Menezes, Patrícia Veras, Natalia Tavares, Pedro M. Moraes-Vieira, Cláudia Ida Brodskyn

**Affiliations:** 1 Instituto Gonçalo Moniz, Fundação Oswaldo Cruz, Salvador, Brazil; 2 University of California San Francisco, Department of Medicine, San Francisco, California, United States; 3 University of Calgary, Departments of Microbiology, Immunology and Infectious Diseases, Calgary, Alberta, Canada; 4 University of Campinas, Department of Genetics, Evolution, Microbiology and Immunology, São Paulo, Brazil; Institute of Postgraduate Medical Education and Research, INDIA

## Abstract

Macrophages represent a fundamental component of the innate immune system that play a critical role in detecting and responding to pathogens as well as danger signals. *Leishmania* spp. infections lead to a notable alteration in macrophage metabolism, whereby infected cells display heightened energy metabolism that is linked to the integrity of host mitochondria. However, little is known about how different species of *Leishmania* manipulate host metabolism. Here, we demonstrate that despite differences in their mechanisms for evading host immune responses, *L*. *amazonensis* and *L*. *braziliensis* induce comparable disruptions in key metabolic pathways. We found that infected macrophages exhibited an overall elevation in energy metabolism regardless of the parasite strain, evidenced by the elevation in glycolysis and oxygen consumption rates, along with increased proton leak and decreased ATP production. We also analyzed the effects of both *Leishmania* spp. strain infection on mitochondria function, further revealing that infected cells display heightened mitochondrial mass and membrane potential. To investigate the metabolic pathways required for *Leishmania* amastigotes to persist in BMDMs, we pre-treated cells with small molecule drugs that target major metabolic pathways, revealing that perturbations in several metabolic processes affected parasite survival in a strain-independent manner. Treatments with inhibitors of the oxidative phosphorylation and glycolysis substantially reduced parasite loads. Collectively, our findings suggest that *L*.*amazonensis* and *L*.*braziliensis* exploit host cell metabolic pathways similarly to survive in macrophages.

## Author summary

Our study delves into the intricate interplay between macrophages and *Leishmania* spp. infections, shedding light on how these parasites manipulate host cell metabolism. Despite variations in the mechanisms employed by different *Leishmania* species to evade immune responses, our findings reveal a striking similarity in their impact on host metabolic pathways. Infected macrophages consistently exhibit heightened energy metabolism, characterized by increased glycolysis and oxygen consumption rates, alongside alterations in mitochondrial function. Importantly, we demonstrate that targeting key metabolic pathways with drugs significantly affects parasite survival, offering promising avenues for therapeutic interventions against leishmaniasis. By elucidating these complex metabolic processes in a clear and accessible manner, our research not only deepens our understanding of host-parasite interactions but also holds significant implications for the development of novel treatment approaches. We aim to bridge the gap between scientific knowledge and its broader implications for both scientists and non-scientists alike, advancing the fight against neglected tropical diseases.

## Introduction

Macrophages comprise a set of innate immune cells that are instrumental in the maintenance of homeostatic processes and the integration of bodily systems [1]. These cells respond to pathogens and danger cues by coordinating both cell-intrinsic and -extrinsic signals [2]. In the settings of infection and inflammation, macrophages undergo a dynamic remodeling of their enzymatic activity to acquire energy and synthesize new building blocks, processes essential for the effective immune responses of these cells [2]. Not surprisingly, intracellular pathogens have developed means to manipulate macrophage metabolic machinery to favor its survival and proliferation [3]. The mounting evidence emphasizing the pivotal role of metabolic processes in shaping the outcome of intracellular pathogen infections has elevated energy metabolism to a central aspect of immune cell biology.

Under resting conditions, macrophages typically rely on oxidative phosphorylation (OXPHOS) of molecules (such as glucose, amino acid, and fatty acids) to meet their energy demands [4]. However, upon inflammatory activation, these cells shift their metabolism from OXPHOS to aerobic glycolysis, which is distinguished by the conversion of glucose into lactate in the presence of oxygen to generate ATP. The process by which macrophages undergo metabolic rewiring from a quiescent state, which relies on OXPHOS-dependent pathways, to an activated phenotype, dependent on glycolysis, is commonly referred to as the Warburg effect [4]. Shreds of evidence suggest that eliminating intracellular pathogens, including viruses, bacteria, fungi, and protozoa depends on this metabolic shift [5,6,7,8]. We, therefore, reasoned that the reorganization of the macrophage metabolic landscape is paramount for elimination of the intracellular trypanosomatids. To further expand on this hypothesis, we leveraged a model of trypanosomatid infection in which bone-marrow-derived macrophages (BMDMs) harvested from C57/Bl6 mice were exposed to either *L*. *amazonensis* or *L*. *braziliensis*. Subsequently, we dissected the metabolic intricacies within these in vitro infection models.

Notably, *Leishmania* spp. is the causative agent of leishmaniasis, a significant health concern affecting millions of individuals, particularly in tropical regions worldwide [9,10]. Abundant evidence supports the notion that *L*. *amazonensis* and *L*. *braziliensis* employ distinct strategies to evade host immune responses, which is evidenced by differences in immunopathological processes. Infections with *L*. *amazonensis* are characterized by a high parasite burden at site of infection, elevated IL-10 production, and an impaired cellular immune response [11]. Conversely, *L*. *braziliensis* infections elicit an intense inflammatory response with the presence of fewer parasites, heightened IFN-γ production, and robust cellular immune responses [11]. Thus, understanding the immunological and metabolic nuances in *Leishmania* spp. infection is imperative for enhancing our knowledge surrounding leishmaniasis and open avenues for better treatment options and patient care. Here, we elucidate key metabolic variances in macrophages infected with *L*. *braziliensis* and *L*. *amazonensis*.

## Materials and methods

### Ethics statement

Female C57Bl/6 mice (*Mus musculus)*, aged 6 to 12 weeks, were kept in the Animal care facility at Gonçalo Moniz Institute / Fiocruz (IGM-FIOCRUZ) in Salvador, Brazil, under controlled conditions and pathogen-free settings. All mouse experiments were performed in accordance with procedures approved by the Ethical Animal Use in experimentation Committee, under protocol number (CEUA/N°006/2018/ IGM / Fiocruz-Bahia).

### Parasite culture

*L*. *amazonensis* (MHOM/Br/00/BA125) and *L*. *braziliensis* (MHOM/BR/01/BA788) strains were used to infect bone marrow-derived macrophages (BMDMs). The promastigotes were maintained in Schneider’s culture medium (Sigma Aldrich, St. Louis, MO) supplemented with 10% fetal bovine serum (Sigma), 2 mM L-glutamine, 100 μg/mL penicillin, and 100 μg/mL streptomycin (Sigma) at 24°C. Throughout the culture period, promastigote counts were performed using a hemocytometer, with a 100x dilution in saline solution (0.9% sodium chloride) to monitor growth dynamics. For all experiments, stationary-phase parasites, enriched in the infective metacyclic promastigotes, were used. The percentage of metacyclic promastigotes in the stationary phase is approximately 70–90% for *L*. *braziliensis* and 60–70% for *L*. *amazonensis* [12].

### *In vitro* bone marrow-derived macrophages differentiation

Bone marrow precursors were harvested by flushing the femurs and tibias of C57BL/6 mouse rear legs with RPMI-1640 medium (Gibco Invitrogen Corporation, Carlsbad, CA, USA) using a 10 mL syringe and a 21 G needle. The collected cells were centrifuged at 1500 RPM for 10 minutes at 4°C. Bone marrow cells were then resuspended in RPMI culture medium (Gibco) supplemented with 10% heat-inactivated fetal bovine serum (Gibco), 2 mM L-glutamine, 100 μg/mL penicillin, 100 μg/mL streptomycin (Gibco), and 30% L929 cell-conditioned medium. The cells were seeded into Petri dishes and incubated for 7 days at 37°C with 5% CO₂ to promote differentiation into macrophages. On the fourth day of culture, the cells were supplemented with complete RPMI medium containing 30% L929 cell-conditioned medium. After differentiation, the adherent BMDMs were harvested using a cell scraper, washed with ice-cold saline solution (0.9% sodium chloride), and centrifuged at 1500 RPM for 10 minutes at 4°C. The cell pellet was resuspended in complete RPMI medium, and the cells were counted using a Neubauer chamber. The cells were then plated at 3×10^5^ cells per well in 24-well plates and incubated for 24 hours to allow macrophage adherence.

### *In vitro* macrophage infection and pharmacological modulation of metabolic pathways

Bone marrow-derived macrophages (BMDMs) were pre-treated with specific metabolic pathway inhibitors for 1 hour before infection, as detailed in S1 Table, and incubated at 37°C with 5% CO₂. Following treatment, the cells were washed with room temperature saline solution (0.9% sodium chloride) to remove any residual inhibitors from the supernatant. Subsequently, the BMDMs were exposed to *L*. *amazonensis* and *L*. *braziliensis* promastigotes at ratios of 5:1 and 10:1, respectively, to ensure comparable infection rates for both *Leishmania* species. After 6 hours of co-incubation, non-internalized parasites were removed by washing with saline solution (0.9% sodium chloride), and the cells were incubated for an additional 12, 18 or 42 hours (corresponding to 18, 24 and 48 hours post infection) at 37°C with 5% CO₂. The cells were then fixed with methanol and stained using hematoxylin eosin kit (LB Laborclin, Brazil). Coverslips were examined under a light microscope (Nikon), and a minimum of 100 cells per experimental condition were counted. The infection index was calculated by multiplying the number of parasites by the percentage of infected cells.

To assess parasite viability, after 24 hours of infection, the cells were incubated in Schneider’s Insect Medium (Sigma Aldrich, St. Louis, MO) at 24°C for 5 days to promote the growth of non-eliminated parasites, which were then counted using a Neubauer chamber.

### Seahorse metabolic analysis

The bioenergetic profile of *L*. *amazonensis* and *L*. *braziliensis*-infected BMDM was analyzed using an XFe-96 extracellular flux analyzer (Seahorse Bioscience). The oxygen consumption rate (OCR) and extracellular acidification rate (ECAR) were measured at the indicated time points post-infection. BMDMs were seeded at 3 × 10^4^ cells/well in 100 μL of RPMI in XFe-96 cell culture plates. After an overnight incubation, the cells were infected with *L*. *amazonensis* and *L*. *braziliensis* metacyclic promastigotes. One hour before the designated infection times, the cells were washed, and the medium was replaced with XF medium (XF RPMI Medium pH 7.4 containing 1 mM HEPES and supplemented with L-glutamine). The bioenergetic profile was measured in real-time under basal conditions and in response to glucose (10 mM), oligomycin (1 μM) and and 2-deoxyglucose (2-DG, 50 mM), for the ECAR evaluation and oligomycin (1 μM), fluoro-carbonyl cyanide phenylhydrazone (FCCP, 2 μM), rotenone (100 nM) and antimycin A (1 μM) for OCR evaluation. Non-glycolytic acidification was determined by selecting the lowest positive value after 2-DG injection. Glycolysis rates were calculated by subtracting non-glycolytic acidification from the highest value post-glucose injection. Glycolytic capacity was determined by subtracting non-glycolytic acidification from the highest value after oligomycin injection, and glycolytic reserve was calculated as the glycolytic capacity minus glycolysis. Non-mitochondrial respiration was assessed by selecting the lowest positive value after rotenone and antimycin A injections. Basal respiration was determined by subtracting non-mitochondrial respiration from basal values. Maximal respiration was assessed by subtracting non-mitochondrial respiration from the values obtained after FCCP injection. Proton leak was calculated by subtracting non-mitochondrial respiration from the values obtained after oligomycin injection. ATP production was assessed by subtracting proton leak from basal respiration rates. The spare respiratory capacity (SRC) was obtained by subtracting the FCCP values from basal OCR values. The procedures used in these experiments followed the manufacturer’s instructions (Seahorse). After the readings on the XFe96, the cells underwent additional protein quantification for data normalization using the Pierce BCA Protein Assay kit (Thermo Scientific).

### Quantification of proteins by BCA kit Pierce reaction

After the XF assays, the cell culture plate was washed once with PBS, and 30 μL of lysis buffer containing 1% NP-40, 50 mM Tris-HCl (pH 7.5), 150 mM NaCl, 1 mM EDTA (pH 8), 10 mM 1,10-phenanthroline, and phosphatase and protease inhibitors (Roche) was added to each well. The plate was then incubated at 4°C under agitation for 20 minutes then frozen and unfrozen. Afterwards, 10 μL of the lysate was transferred to a new plate for protein quantification using the Pierce BCA kit assay. Following the addition of the reagent A+B mix to the samples, the plate was incubated for 30 minutes at 37°C and then read by spectrophotometry at 562 nm, according to the manufacturer’s instructions. The quantification results were subsequently uploaded into the Wave Desktop software, where they were normalized by the software.

### Mitochondrial markers and reactive oxygen species content assessment

Mitochondrial function was assessed by analyzing membrane potential and mitochondrial mass. BMDMs were seeded in polypropylene round-bottom tube (FALCON) at a density of 1 × 10^6^ cells per tube. After pre-treating the cells with metabolic inhibitors, the BMDMs were washed and infected with *L*. *amazonensis* and *L*. *braziliensis*. Then, the BMDMs were washed with 1x PBS, and the cells were transferred to a 96-well plate. They were then incubated with a mix (50 μL per well) of MitoTracker Green (50 nM) (Invitrogen), MitoTracker Red (125 nM) (Invitrogen), anti-mouse F4/80 (BioLegend), and Live/Dead (eBioscience) dye for 30 minutes at 37°C in the dark. After staining, the cells were washed with 100 μL of 1x PBS + 2% FBS and centrifuged at 1500 rpm for 5 minutes. The supernatant was discarded, and the cells were resuspended in 200 μL of 1x PBS + 2% FBS for flow cytometry analysis. Samples were acquired using FACSDIVA software (BD Biosciences) and analyzed with FLOWJO software (BD).

Reactive oxygen species (ROS) production was measured using the CellROX probe. BMDMs were seeded in 24-well plates at a density of 5 × 10^5^ cells per well, pre-treated with metabolic inhibitors, and infected with *L*. *amazonensis* and *L*. *braziliensis*. Afterwards, cells were incubated with CellROX Green reagent (5 μM) for 30 minutes at 37°C with 5% CO₂, then washed three times with 1x PBS (Gibco), fixed with 3% paraformaldehyde (PFA), and stained with DAPI antifade ProLong Gold (Thermo Fisher Scientific, IL, USA). Fluorescence microscopy (Leica DMi8) with 485/520 nm excitation was used to capture images. Approximately 30 cells per experimental condition were selected and analyzed, measuring area, integrated density, mean gray value, and median. Measurements were also taken in non-fluorescent areas adjacent to the cells. The obtained values were analyzed to yield the Corrected Total Cell Fluorescence (CTCF), calculated as: CTCF = Integrated density—(selected cell area × mean fluorescence of background), using ImageJ software (National Institutes of Health, USA).

### Determination of the intracellular parasite load

At 18, 24 and 48 hours post-infection, slides containing pre-treated and infected macrophages were fixed with methanol and stained using the hematoxylin-eosin kit (LB Laborclin, Brazil). Coverslips were examined under a light microscope (Nikon). To evaluate the infection rate, a minimum of 100 cells per experimental condition were counted. The parasite load within the macrophages was determined by counting the amastigotes present in each infected cell out of the total number of cells counted across the various experimental conditions. The infection index was calculated by multiplying the number of parasites by the percentage of infected cells.

### Determination of parasite binding and phagocytosis activity in infected Bone marrow- derived macrophages

Bone marrow-derived macrophages (BMDMs) were pre-treated with specific metabolic pathway inhibitors for 1 hour prior to infection and incubated at 37°C with 5% CO₂. After treatment, the cells were washed with room-temperature saline solution (0.9% sodium chloride) to remove any residual inhibitors. BMDMs were then exposed to *L*. *amazonensis* and *L*. *braziliensis* promastigotes at ratios of 5:1 and 10:1, respectively, and centrifuged at 300g for 5 minutes at 4°C. For binding analysis, the plate remained in the centrifuge for an additional 25 minutes, after which the cells were washed with cold saline, fixed with methanol, and stained with eosin and hematoxylin. For phagocytosis analysis, following the initial 5-minute centrifugation, the plate remained in the centrifuge for another 25 minutes. Afterward, the cells were washed with cold saline, the medium was replaced with RPMI at 37°C, and incubation continued for 2 additional hours at 37°C with 5% CO₂. The cells were then fixed with methanol and stained using a hematoxylin-eosin kit (LB Laborclin, Brazil). Coverslips were examined under a Nikon light microscope, and a minimum of 100 cells were counted for each experimental condition

### Parasite growth curve and parasite viability after infection

For growth curve analysis, metabolic pathway inhibitors were added individually to the cultures of *L*. *amazonensis* and *L*. *braziliensis* for 1 hour. After this period, the culture was centrifuged with cold saline at 3000 rpm for 10 minutes at 4°C and resuspended in Schneider’s medium. Daily counts of the parasite cultures were performed for 4 days. For the viability of the parasites, BMDMs were treated with inhibitors for 1 hour either before or after infection, and the infection lasted 24 hours with *L*. *amazonensis* or *L*. *braziliensis*. After the infection period, the RPMI medium was replaced with Schneider’s medium, and cultivation continued for an additional 5 days to count the viable parasites.

### Statistical analysis

Statistical analysis was conducted using GraphPad Prism v.10 (GraphPad Software, La Jolla, CA) and R statistical software, version 4.31. Packages utilized for the generation of plots include ggplot2 and tidyverse. The analyzed data followed a non-parametric distribution pattern. The analyses were performed using the Kruskal-Wallis test, followed by the Dunn post-test, and the values were expressed as the median with interquartile deviation. Differences were considered significant when p < 0.05, using a 95% confidence interval.

## Results

### 01- *Leishmania* spp. infection promotes metabolic rewiring in bone-marrow derived macrophages

**Fig 1:**
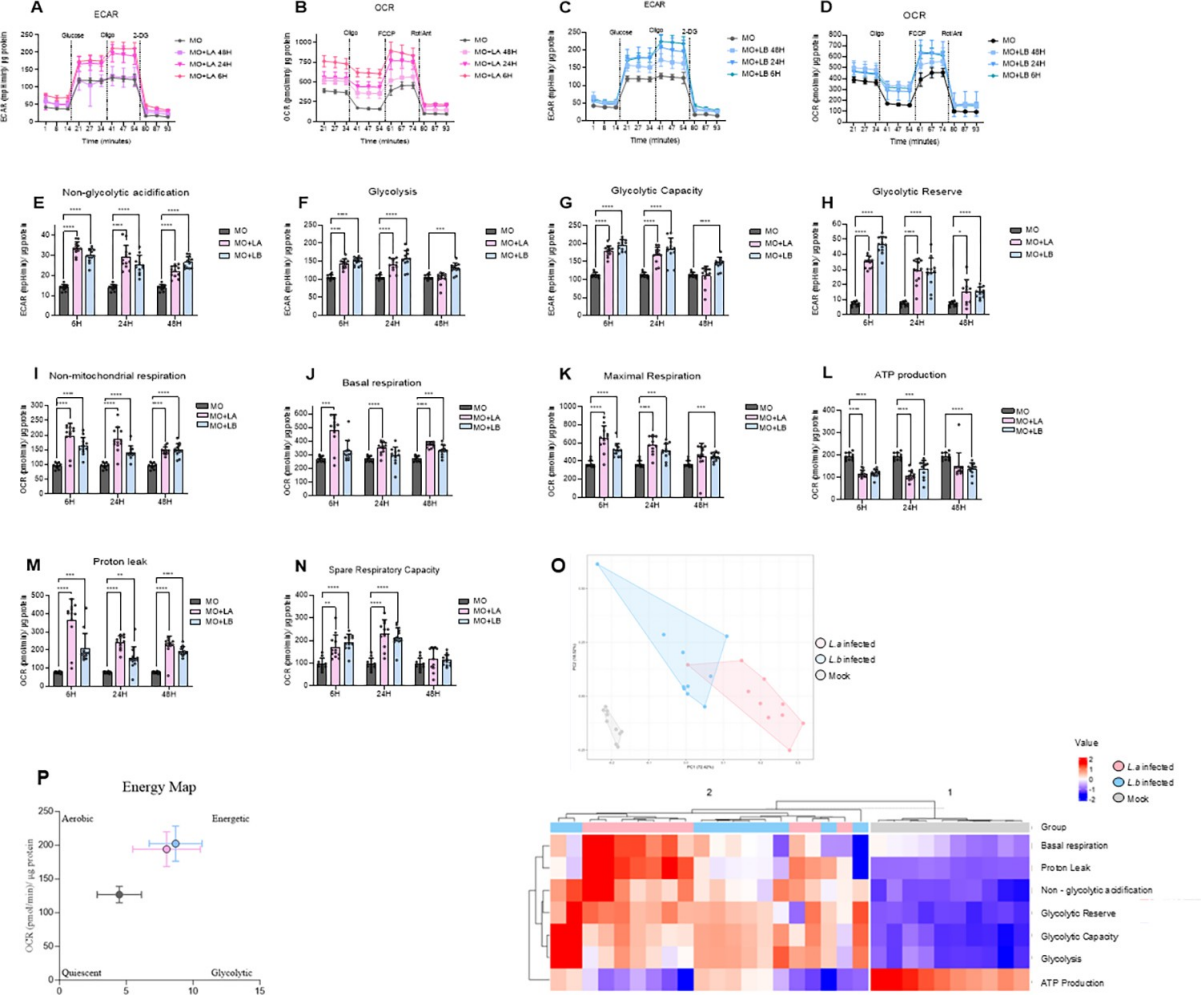
Assessment of metabolic alterations in Leishmania-infected bone marrow-derived macrophages. A) Real-time extracellular acidification rate (ECAR) analysis of bone marrow-derived macrophages from uninfected (mock) C57BL/6 mice, infected with La (*Leishmania amazonensis*) for 6 hours, and then incubated for an additional 18- and 42-hours post-infection. B) Real-time oxygen consumption rate (OCR) analysis of bone marrow-derived macrophages from uninfected (mock) C57BL/6 mice, infected with La (*Leishmania amazonensis*) for 6 hours, and then incubated for an additional 18- and 42-hours post-infection. C)Real-time extracellular acidification rate (ECAR) analysis of bone marrow-derived macrophages from uninfected (mock) C57BL/6 mice, infected with Lb (*Leishmania braziliensis*) for 6 hours, and then incubated for an additional 18- and 42-hours post-infection. D) Real-time oxygen consumption rate (OCR) analysis of bone marrow-derived macrophages from uninfected (mock) C57BL/6 mice, infected with Lb (*Leishmania braziliensis*) for 6 hours, and then incubated for an additional 18- and 42-hours post-infection. E) Non-glycolytic acidification, F) Glycolysis, G) Glicolytic capacity, H) Glicolytic reverse, I) Non- mitochondrial respiration, J) Basal respiration, K) Maximal respiration, L) ATP-production, M) Proton leak, N) Spare respiratory capacity, O) Principal component analysis (PCA) and Heatmap of observed alterations in metabolic reprogramming, P) Energy map.Two independent experiments were conducted, each with five replicates, using two different cell lots. Statistical analysis was performed using the 2-way ANOVA test with Dunn’s post-test. ***<0,0001 **p < 0.01, *p < 0.05.

To assess the impact of *Leishmania* spp. infection on macrophage metabolism, we exposed BMDMs to either *L*. *braziliensis* or *L*. *amazonensis* promastigotes for 6 hours and subsequently incubated the infected BMDMs for more 18 and 42 hours, corresponding to a total time of 24 and 48 hours. Subsequently, we quantified metabolic changes by measuring extracellular acidification (ECAR) and oxygen consumption rates (OCR) after 6, 24 and 48 hours of infection. On one hand, both *Leishmania*-infected groups exhibited a notable increase in both ECAR and OCR at 6 and 24 hours after infection when compared to uninfected controls (Fig 1A, 1B, 1C and 1D). The increase in OCR in infected macrophages may be attributed to oxygen consumption by both the macrophages and *Leishmania*. However, distinguishing between the two bioenergetic contributions is not possible due to limitations in the Seahorse extracellular flux analyzer system. On the other hand, at 48 hours after infection, it was detected a metabolic profile similar to uninfected macrophages (Fig 1A, 1B, 1C and 1D).

This metabolic alteration was comparable between parasite strains. Expanding our investigation into the modulation of macrophage metabolic capacities by *Leishmania* spp., we further characterized the metabolic landscape in our cell cultures. Our data indicate that *Leishmania* infection led to a significant elevation in basal glycolysis, glycolytic capacity, maximal respiration rates and proton leak (Fig 1F, 1G, 1K and 1M). Conversely, *Leishmania* spp. infection resulted in reduced ATP production (Fig 1L). To gain a comprehensive perspective on the influence of parasite infection on key metabolic parameters measured by ECAR and OCR, we applied principal component analysis (PCA)and a heat map of the results obtained in the SeaHorse analysis This analytical approach consistently confirmed a parasite-induced metabolic reprogramming in BMDMs, irrespective of the parasite strain (Fig 1O). Furthermore, our energy map illustrates that both *L*. *amazonensis* and *L*. *braziliensis* can shift the metabolic profile of infected cells toward a higher-energy state, as indicated by the concurrent enhancements in both ECAR and OCR, when compared to metabolically quiescent uninfected macrophages (Fig 1P). In summary, these findings support that infection with both *Leishmania* parasite strains results in the metabolic rewiring of infected BMDMs.

### 02- Impact of *Leishmania* spp. infection on mitochondrial function

**Fig 2:**
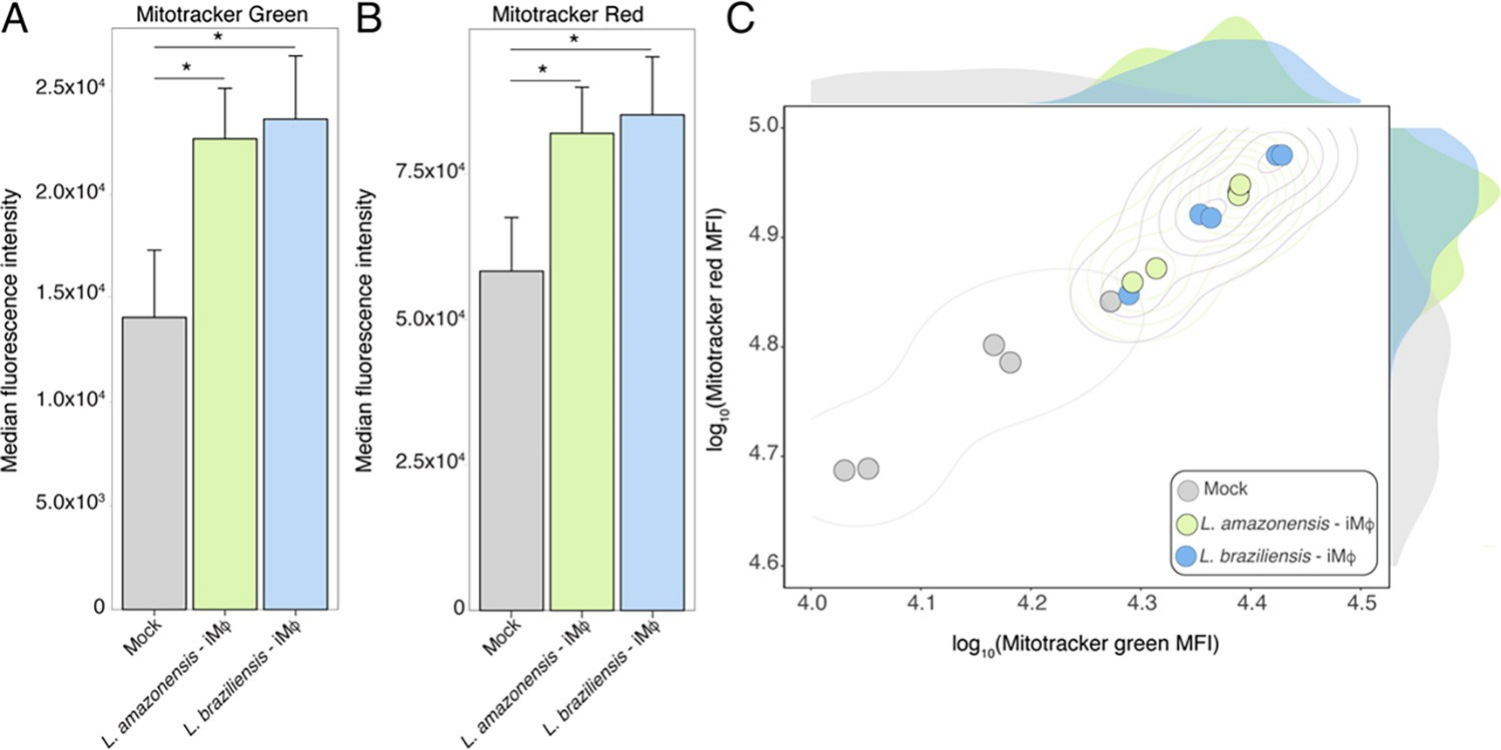
*Leishmania* spp promote increased mitochondrial mass and membrane potential. After the infection, BMDMs were incubated for 30 minutes with 50nM of MitoTracker Green probe (A) and 125 nM of MitoTracker Red probe (B), for analysis of mitochondrial mass (MM) and mitochondrial membrane potential (MMP), respectively. Samples were acquired using the FACSDIVA software (BDBiosciences) and analyzed using FLOWJO Software (BD). (C) correlation between MitoTracker green and MitoTracker Red with infected macrophages. The experiments were performed in triplicate with five replicates per experiment. Statistical analysis was conducted using the Kruskal-Wallis test with Dunn’s post-test, with a significance threshold of *p < 0.05.

Recognizing the pivotal role of mitochondria in meeting cellular energy demands and orchestrating responses to disruptions in cellular homeostasis, we speculated that *Leishmania* spp. infection alters mitochondria function. We evaluated the mitochondria mass (MM) content and membrane potential (MP) using commercially available mitochondrial probes (MitoTracker green and red, respectively). Our findings indicate a substantial increase in both mitochondria mass (Fig 2A) and membrane potential (Fig 2B) in BMDMs infected with *Leishmania* parasites. Notably, we observed a strong positive correlation between these markers, with infected macrophages displaying a notably elevated profile compared to their non-infected counterparts (Fig 2C). These results underscore the significant influence of *Leishmania* spp. infection on mitochondria functionality within the host cells.

### 03- Pharmacological targeting of macrophage metabolic reprogramming mitigates *Leishmania* spp. infection

Next, we sought to understand which metabolic pathways are required to support *Leishmania* amastigote persistence in BMDMs. Therefore, we pre-treated cells with modulators of metabolic pathways for 1 hour prior to exposure to parasites. Notably, the drugs used in our assay targeted key metabolic pathways in host cells, including glycolysis, OXPHOS, and fatty acid metabolism (detailed in S1 Table). The doses of the inhibitors of metabolic pathways were chosen considering the maintenance of cellular viability of the macrophages, as shown in S2 Fig. Subsequently, as a proxy for the metabolic influence on both susceptibility and permissibility to *Leishmania* spp. infection, we assessed the percentages of infected cells and the number of viable amastigotes within pre-treated BMDMs.

**Fig 3:**
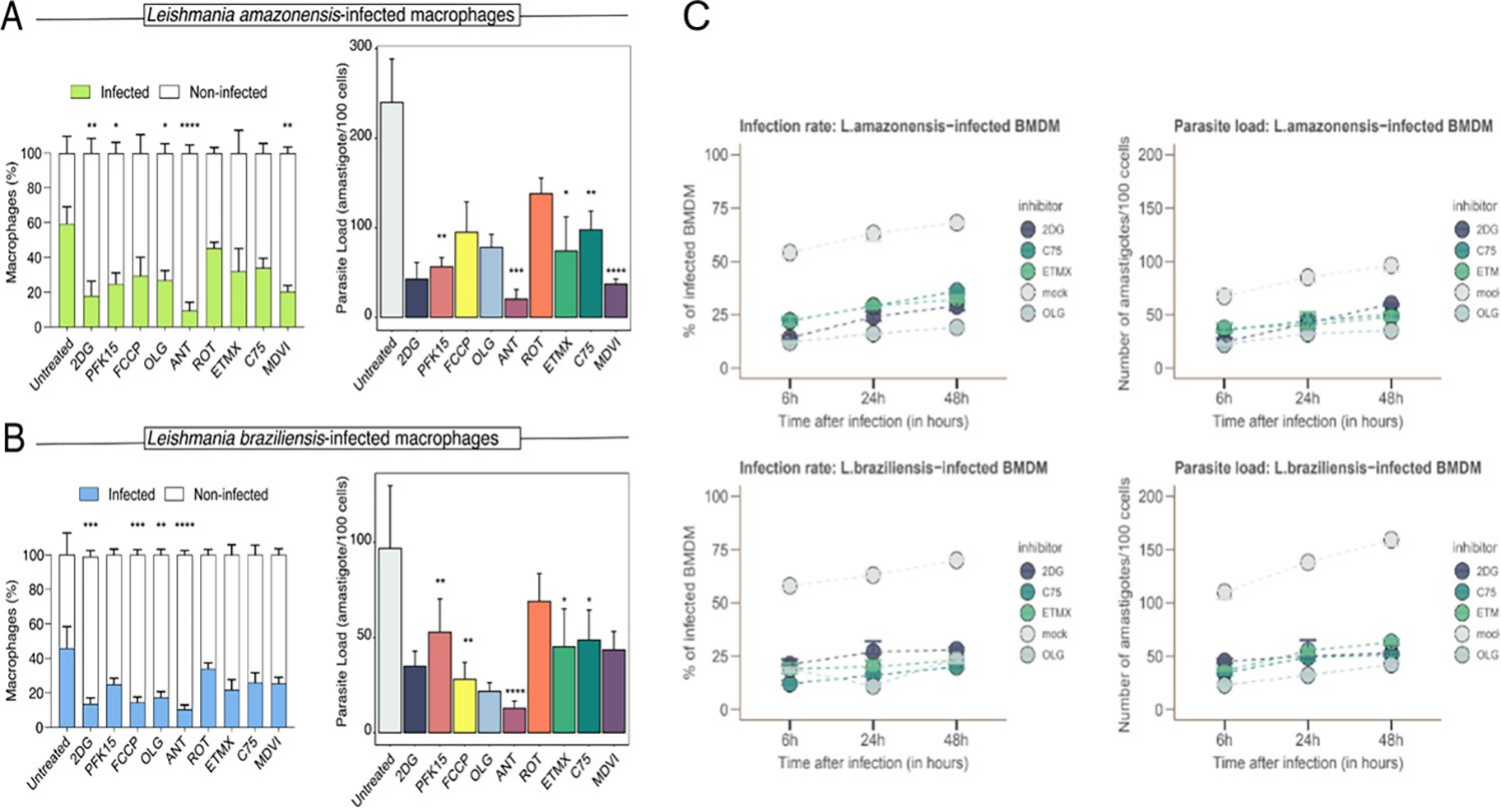
Evaluation of infection rate and parasite load in macrophages treated with metabolic inhibitors during *L*. *amazonensis* or *L*. *braziliensis* infections. Infection rate and parasite load of BMDMs treated with metabolic inhibitors. BMDMs were treated with the inhibitors 1 hour before infection for 6 hours and then incubated for a further 12 hours. a) Infection rate and parasite load of BMDMs by *L*. *amazonensis* under the influence of metabolic pathway inhibitors. b) Infection rate and parasite load of BMDMs infected with *L*.*braziliensis* under the influence of metabolic pathway inhibitors representing the number of internalized amastigotes in 100 cells. 2DG, 2-deoxyglucose; PFK15, Phosphofructokinase; FCCP, Phenylhydrazone; OLG, Oligomycin; Ant A, Antimycin, ROT; Rotenone, ETMX, Etomoxir, C75; MDVI. c) Longitudinal Analysis of the Impact of Metabolic Inhibitors on *Leishmania spp*. *Infection* in BMDMs. 2DG, 2-deoxyglucose; C75, ETMX, Etomoxir, OLG, Oligomycin. The experiments were performed in triplicate with five replicates per experiment. Statistical analysis was conducted using the Kruskal-Wallis test with Dunn’s post-test. ****p <0.0001, ***p <0. 001, **p < 0.01, *p < 0.05.

Overall, perturbations in several metabolic processes affected the survival of both parasite strains (Fig 3A and 3B). Treatment with 2DG, which inhibits the glucose flux, resulted in a decreased percentage of macrophages infected with parasites compared to untreated infected macrophages. Additionally, we observed a decrease in the percentage of *L*. *amazonensis*-infected cells treated with PFK15, a small molecule inhibitor targeting PFKFB3 an enzyme pivotal in glycolysis though this effect was not observed in *L*. *braziliensis*-infected cells. Moreover, PFK15 treatment led to a reduction in the number of amastigotes for both parasite strains.

We noted that the mitochondria complex III inhibitor antimycin had the most significant impact on parasite load in both infection models. Also, inhibition of mitochondrial fission (MDVI treatment) resulted in a marked reduction in parasite load only in cells infected with *L*. *amazonensis*. Additionally, we found that uncoupling of mitochondrial membrane potential (treatment with FCCP) only reduced the number of *L*. *braziliensis* amastigotes. These observations may point to differences in how these *Leishmania* species exploit host mitochondrial metabolism to support parasite survival. Blocked mitochondrial complexes I with rotenone did not attenuate parasite loads. Interfering with fatty acid metabolism substantially impacted the survival of both species, as indicated in cells treated with low doses of etomoxir and C75 (inhibitors of fatty acid oxidation and synthesis, respectively). We observed that both the infection rate and the parasitic load in infected macrophages slightly increased at 6, 24, and 48 hours post-infection in BMDMs infected with any of both species of *Leishmania*. The addition of 2DG, C75, Etomoxir, and oligomycin significantly reduced these parameters (Fig 3C). In conclusion, our findings reveal intricate metabolic nuances underlying *Leishmania* spp. infection and shed light on how different strains exploit host cell energy metabolism to survive within macrophages.

### 04- Assessment of most effective metabolic inhibitor to mitigate *in vitro Leishmania* spp. infection

**Fig 4:**
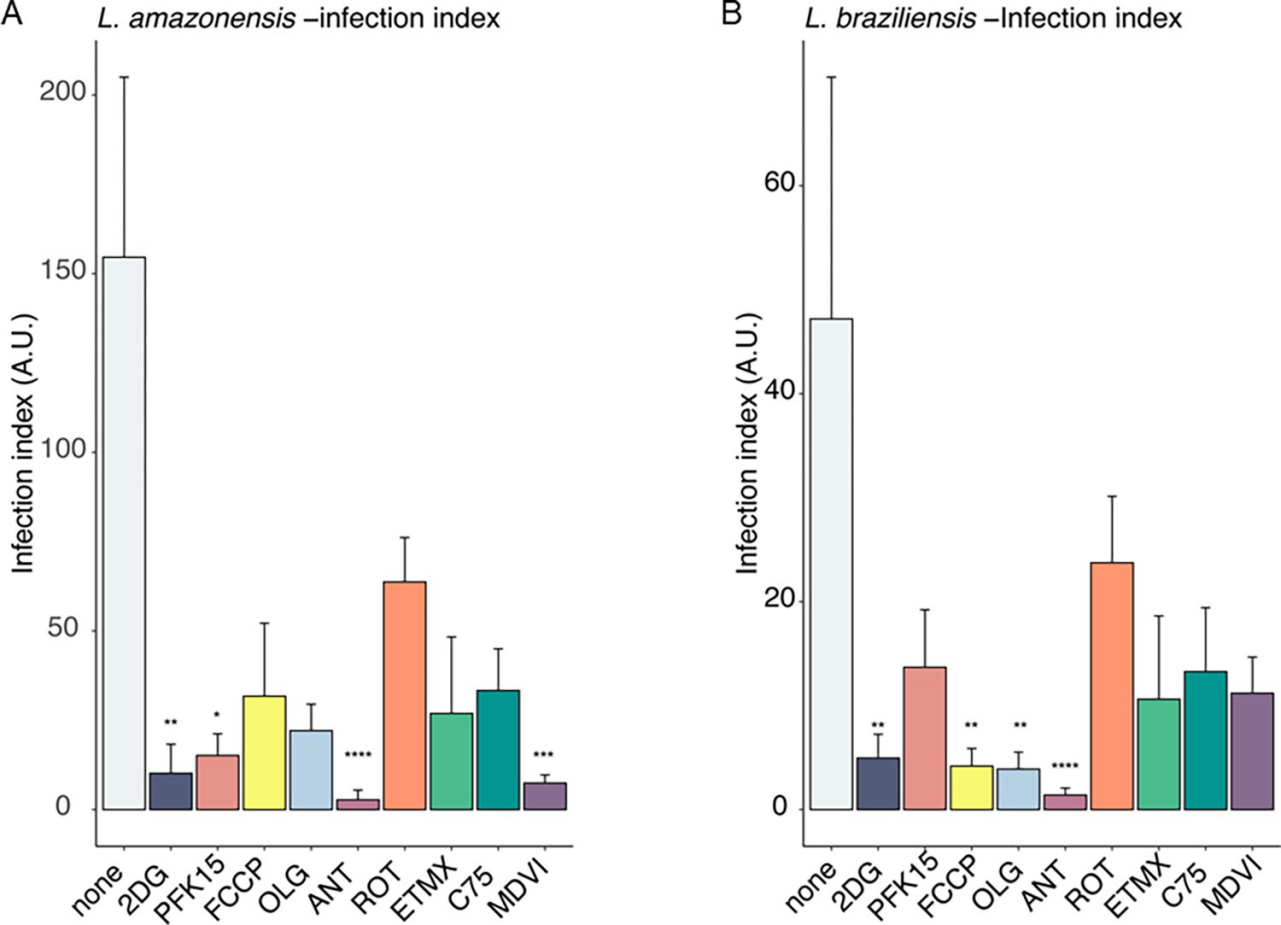
Determination of infection index following metabolic inhibitor treatment. The infection index is determined by multiplying the percentages of infected cells by the number of parasites encountered in each well, thereby assigning equal weight to both infection rates and parasite load. a) *L*. *amazonensis* infection and b) *L*. *braziliensis* infection using different metabolic inhibitors 2DG, 2—Deoxyglucose; PFK15, Phosphofructokinase; FCCP, Phenylhydrazone; OLG, Oligomycin; Ant A, Antimycin, ROT; Rotenone, ETMX, Etomoxir, C75; MDVI. The experiments were performed in triplicate with five replicates per experiment. Statistical analysis was conducted using the Kruskal-Wallis test with Dunn’s post-test. ****p <0.0001, ***p <0. 001,**p < 0.01, *p < 0.05.

To assess the overall extent of infection in our experimental model of *L*. *amazonensis* and *L*. *braziliensis* infection, we calculated the infection index. This index is determined by multiplying the percentages of infected cells by the number of parasites encountered in each well, thereby assigning equal weight to both infection rates and parasite load. In our analysis, treatment with 2DG and antimycin A consistently reduced the infection index in both parasite strains (as shown in Fig 4A and 4B). Intriguingly, we observed that PFK15 and MDVI treatments selectively lowered the infection index in *L*. *amazonensis*-infected macrophages, while FCCP and oligomycin had the same effect in *L*. *braziliensis*-infected cells. These findings collectively suggest strain-specific dependencies on distinct metabolic pathways for the survival of these parasites within host cells.

To further validate our findings, we used metabolic inhibitors to assess their impact on the growth of *L*. *amazonensis* and *L*. *braziliensis* in axenic cultures, as well as on the viability of intracellular parasites. S2A Fig shows that 2DG does not affect parasitic growth in axenic cultures of either *Leishmania* species. In contrast, oligomycin and antimycin significantly reduced parasite numbers after 3 days of culture, which is expected given *Leishmania’s* reliance on mitochondrial metabolism for proliferation [13]. These two compounds also decreased the number of viable parasites, although this effect was observed after 5 days of culture, following the replacement of RPMI with Schneider medium (S3A Fig). S3A Fig illustrates the different treatment periods with metabolic inhibitors before and after infection, respectively. Despite these treatments, a considerable number of parasites remained viable, and it is important to note that all of our analyses were conducted within 48 hours, a time frame in which the inhibitors would likely affect the metabolism of the infected macrophages rather than directly impact *Leishmania*.

### 05-Impact of metabolic reprogramming on the killing of parasites during phagocytosis

**Fig 5:**
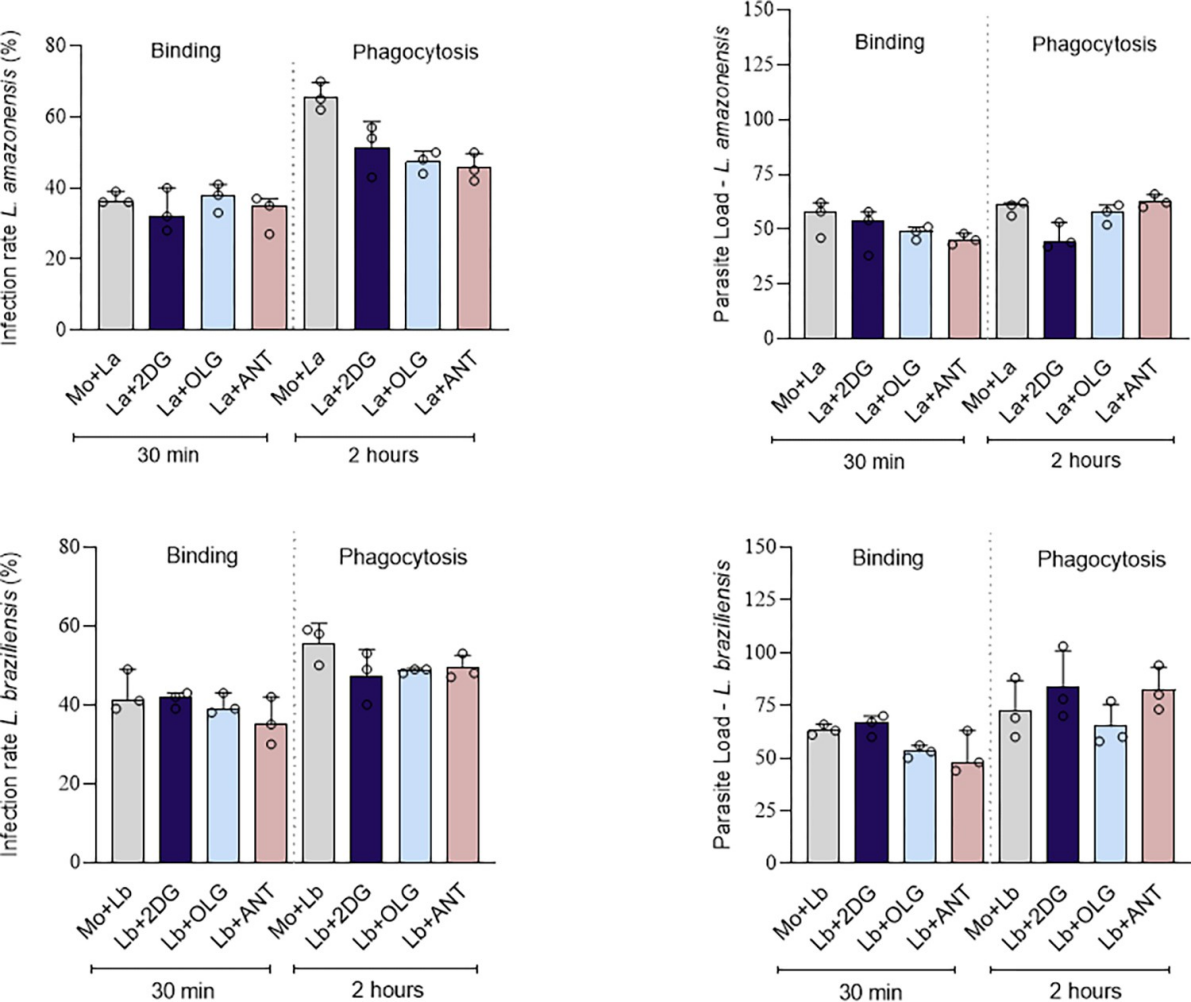
Inhibitor metabolic pathways did not affect binding and phagocytosis of the parasites. BMDMs were treated with the inhibitors 1 hour before infection and then infected for 30 minutes for binding and 2 hours for phagocytosis. A) Infection rate and parasite load of BMDMs by *La*—*L*. *amazonensis*. B) Infection rate and parasite load of BMDMs infected with *Lb—L*.*braziliensis*. 2DG, 2-deoxyglucose; OLG, Oligomycin; Ant, Antimycin. The experiments were performed in triplicate. Statistical analysis was conducted using the Kruskal-Wallis test with Dunn’s post-test. *p < 0.05.

To determine whether metabolic pathway inhibitors influence parasite death during phagocytosis, we pre-treated macrophages with these inhibitors before infection. We then assessed binding and phagocytosis rates to evaluate any potential alterations caused by these compounds. As shown in Fig 5, no differences were observed among the experimental conditions, suggesting that the metabolic pathway inhibitors neither induced parasite killing at early post-infection time points nor interfered with the phagocytosis pro**c**ess.

### 06- Treatment with 2-Deoxy-6-glucose and Antimycin A amplifies superoxide production in infected macrophages

**Fig 6:**
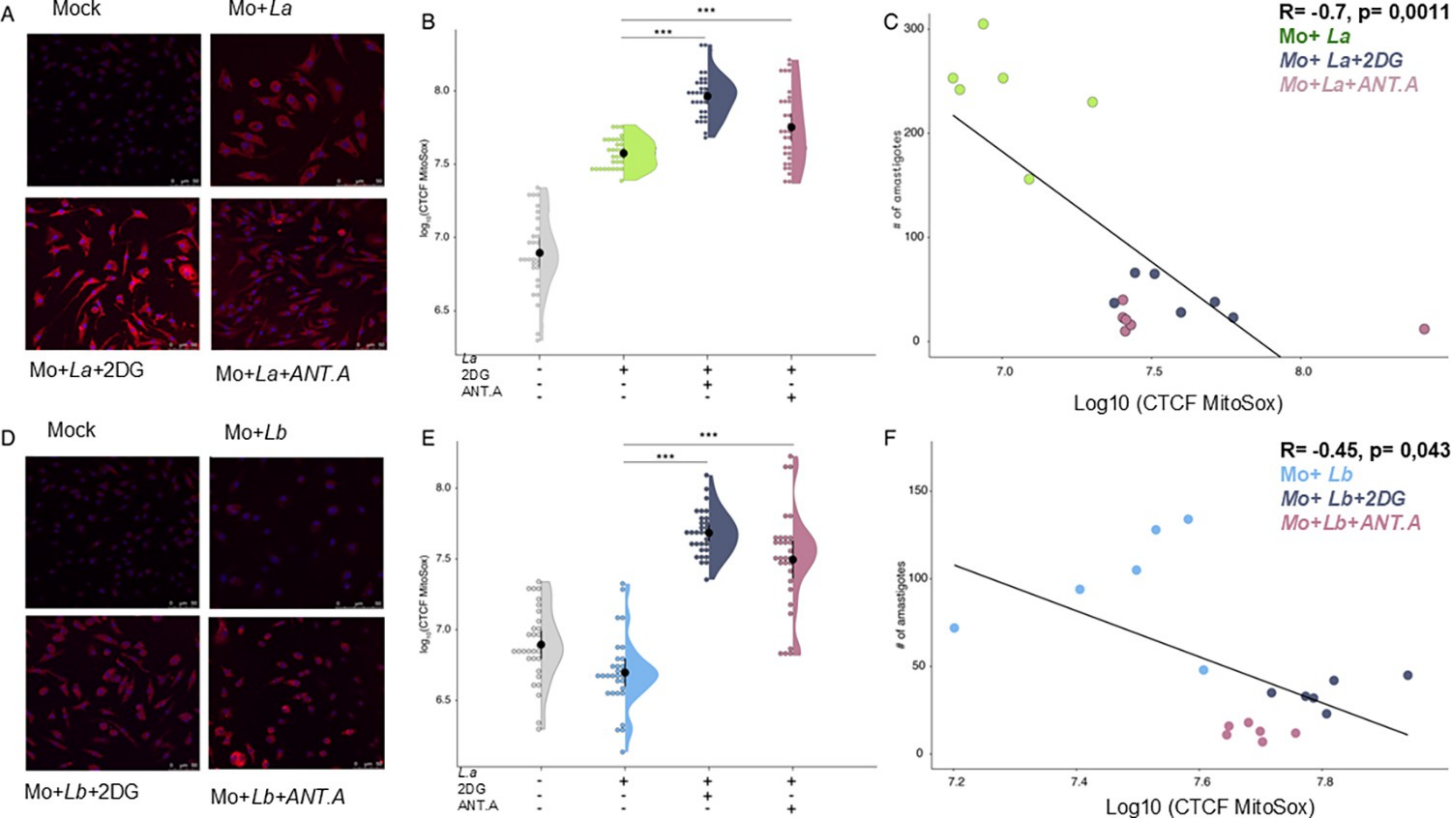
Effects of 2DG and Antimycin A treatment on ROS levels and *Leishmania* parasite load in infected macrophages. To investigate the hypothesis that metabolic inhibitor treatment induces an increase in ROS levels, we evaluated superoxide production in infected BMDS treated with 2DG and Antimycin A using the MitoSOX probe. Total Cellular Corrected Fluorescences (CTCF) were analyzed and quantified for approximately 30 cells per experimental condition. a) Confocal microscopy images depict the experimental groups. b) Scatterplots illustrate the levels of ROS detected via the MitoSOX probe. c) Spearman correlations were performed to analyze the relationship between the number of amastigotes in 100 macrophages (parasite load) and ROS levels. Kruskal-Wallis test with Dunn post-test. ***p <0.001.

It is widely recognized that both pathogen-associated molecular patterns (PAMPs) and the immunometabolic status play pivotal roles in shaping the redox environment within immune cells [12]. Moreover, there is evidence suggesting that the augmentation of reactive oxygen species (ROS) levels could serve as a potential mechanism for eliminating *Leishmania* spp. in infected macrophages [14]. In our study, we aimed to elucidate the mechanistic underpinnings of *Leishmania* elimination in macrophages treated with metabolic inhibitors. To achieve this, we assessed the levels of hydrogen peroxide in mitochondria utilizing the commercially available probe MitoSox. Our findings revealed that treatment with 2DG and Antimycin A, known metabolic inhibitors with a high potential for reducing parasite loads, led to increased levels of mitochondria-derived ROS in both *L*. *amazonensis*- and *L*. *braziliensis*-infected macrophages (Fig 6A, 6B, 6D, and 6E). Furthermore, we observed a negative correlation between ROS levels and the number of amastigotes present in macrophages, suggesting that the elevation of ROS induced by metabolic perturbation impairs parasite survival (Fig 6C and 6F). Taken together, these findings indicate a potential connection between immunometabolic perturbation, remodeling of the redox landscape, and the persistence of parasites in infected macrophages.

## Discussion

When encountering pathogens, quiescent macrophages rapidly adjust their metabolic processes to orchestrate immune functions and respond to infections [4]. However, over the course of evolution, intracellular pathogens have developed strategies to manipulate host metabolism, forming a crucial component of their tactics to evade immune responses and, consequently, to persist within the host [14,15,16,17]. Observations from various studies suggest that macrophages serve as a preferred niche for intracellular protozoa such as *Leishmania* spp., facilitating their differentiation and proliferation [18]. This notion finds support through multiple approaches grounded in metabolic and RNA-sequencing analyses, which consistently highlight macrophages as primary sources of essential metabolites for *Leishmania* spp. [19].Nevertheless, the extent to which distinct *Leishmania* species vary in their impact on macrophage metabolic programs remains a topic that lacks comprehensive understanding.

The present study reveals that both *L*. *amazonensis* and *L*. *braziliensis* infections enhance the energy metabolism of BMDMs, as indicated by increased ECAR and OCR (after 24 and 48 hours of promastigote to amastigote differentiation) In light of this, we conducted experiments at 6, 24, and 48 hours post-infection to observe the metabolic profiles of macrophages infected with *Leishmania amazonensis* or *Leishmania braziliensis*. The metabolic profiles at 6 and 24 hours post-infection were found to be very similar. However, at 48 hours post-infection, we observed a reduction in metabolic activity, as measured by ECAR and OCR, in infected macrophages for both *Leishmania* species. Notably, at 48 hours, *L*. *amazonensis*-infected macrophages exhibited metabolic activity levels comparable to those of non-infected macrophages in the ECAR evaluation. These results suggest that following differentiation into amastigotes, the host cell behaves similarly to a non-infected macrophage, at least concerning *L*. *amazonensis* infection. We can hypothesize that the metabolic shift occurs during the early events of infection, specifically during the differentiation of parasites from promastigotes to amastigotes, as previously demonstrated by Moreira et al. and Ospina et al. with *L*. *infantum* and *L*. *donovani* [20,21].

These findings align with previous observations regarding the influence of other *Leishmania* species on macrophage metabolism. For instance, a study by Ty and colleagues demonstrated that *L*. *amazonensis* and *L*. *donovani* upregulate OXPHOS in human monocyte-derived macrophages [22]. Similarly, Moreira *et al* showed that *L*. *infantum*-infected macrophages shift from glycolysis to OXPHOS in a SIRT1-AMPK dependent manner [20].

In our study, *Leishmania* infection resulted in decreased ATP production related to oxidative phosphorylation (OXPHOS) and an increase in maximum respiration. We propose that *L*. *amazonensis* may cause this ATP reduction by redirecting or depleting host cell energy sources, thereby compromising energy production capacity in host cells. It is important to note that ATP also functions as a signaling molecule [23], and the decrease in ATP levels might disrupt various intracellular signaling pathways, potentially impacting macrophage immune functions. However, it is worth considering that the intracellular ATP pool may be maintained at a stable level due to compensatory ATP production from glycolysis. Further investigations are necessary to accurately measure the ATP pool size in *Leishmania* infection and qualitatively assess its role in influencing resistance to the infection.

Mitochondria plays a pivotal role in diverse cellular processes. In our study, we observed that infection with *L*. *amazonensis* and *L*. *braziliensis* led to a significant increase in mitochondrial mass and membrane potential. Previous studies have reported that *L*. *donovani*-infected macrophages experience a substantial loss of mitochondrial potential and a reduced proximity of mitochondrial-endoplasmic reticulum contact sites [24]. Notably, Ospina *et al* demonstrated that the upregulation of oxidative phosphorylation (OXPHOS) and mitochondrial biogenesis during *L*. *donovani* infection is contingent on the virulence factor glycolipid lipophosphoglycan (LPG), which induces the expression of key transcription factors related to mitochondrial characteristics [21]. These same researchers also found that enhanced mitochondrial biogenesis is associated with a more pronounced infection in macrophages. In our experiments, targeted inhibition of mitochondrial fission resulted in a reduction in *L*. *amazonensis* infection. This suggests the parasite reliance on a well-maintained mitochondrial environment for its successful survival and proliferation.

The reduced infection rate and parasite load in macrophages treated with inhibitors targeting metabolic pathways shed light on the intricacies of how the studied *Leishmania* species persist within host cells.

It is evident that the heightened glycolysis in macrophages significantly contributes to their capacity for increased pro-inflammatory cytokine production, including interleukin-1 beta (IL-1β), thus reinforcing their effectiveness in eradicating intracellular pathogens [4]. Furthermore, research has shown that the ability of neutrophils to curtail the persistence of *L*. *donovani* depends on their enhanced glycolytic activity, suggesting that the induction of glycolysis is a shared antileishmanial mechanism among myeloid cells [25]. Once glycolysis was inhibited with PFK15, the number of amastigotes found in macrophages was significantly reduced. This prompts the hypothesis that the accumulation of glycolytic intermediates plays a central role in *Leishmania* replication within macrophages, which could explain the decrease in parasite load, even if it coincides with a reduction in pro-inflammatory cytokine release by macrophages.

Inhibition of fatty acid synthesis and oxidation with C75 and etomoxir, respectively, also led to a decrease in parasite load and the infection rate. Fatty acid metabolism is a crucial intracellular process that contributes to energy production, cell structure, cell signaling, and various physiological functions [26]. Rabhi *et al* demonstrated that *L*. *major*-infected macrophages can accumulate lipid droplets, which are subsequently utilized by *L*. *major* as high-energy sources [27]. Mitochondrial complex III, also known as the cytochrome bc1 complex or the ubiquinol-cytochrome c oxidoreductase, is a vital component of the mitochondrial electron transport chain [28] and the inhibition of complex III with Antimycin A decreased parasite loads without affecting macrophage viability. However, it is important to recognize that this effect may also be attributed to a direct impact on the mitochondrial fitness of *Leishmania*, as Smilkstein et al. demonstrated that Antimycin A treatment eliminated *Plasmodium spp*. parasites in in vitro assays [29]. Indeed, both oligomycin and antimycin resulted in a reduction in the number of promastigotes in axenic cultures, as well as a decline in parasite viability. Notably, these decreases were observed after 72 hours and 5 days, respectively, which supports the notion that mitochondria play a crucial role in the growth of the parasite within infected cells. The decrease in parasites observed in our assays does not correlate with destruction or reduction of binding, as demonstrated in Fig 5, supporting the idea that the metabolism of the infected macrophage contributes to the proliferation of *Leishmania spp*.

Significantly, treatment with 2DG and Antimycin A not only increased ROS levels but also showed a negative correlation with parasite load in both infection models. This suggests that the treatment-induced alteration of the redox landscape in infected macrophages may play a pivotal role in reshaping the intracellular microenvironment, thereby potentially impacting *Leishmania* survival dynamics.

To the best of our knowledge, this study is important since it investigated the application of a library of small molecules that target key metabolic pathways in an *in vitro* model of *L*. *amazonensis* and *L*. *braziliensis* infection. Nevertheless, a notable limitation in our research lies in the absence of functional assays that could establish a clear link between the metabolic states of macrophages and their effector functions responsible for *Leishmania* elimination. Put together, our findings have identified crucial metabolic pathways essential for *Leishmania* persistence within macrophages. This highlights the intricate interplay between host cell metabolic processes and *Leishmania* survival strategies within macrophages, underscoring the significance of comprehending these dynamics for the development of effective interventions against *Leishmania* infections.

## Supporting information

S1 Fig Assessment of metabolic alterations in Leishmania-infected bone marrow-derived macrophages comparing different infection time points. Real-time extracellular acidification rate (ECAR) analysis of bone marrow-derived macrophages from uninfected (mock) C57BL/6 mice and those infected with *La* (*Leishmania amazonensis*) or *Lb* (*Leishmania braziliensis*) for 6 hours, followed by incubation for an additional 18 or 42 hours post-infection. Real-time oxygen consumption rate (OCR) analysis was also performed under the same conditions. This data highlights metabolic differences in macrophages at distinct infection time points. A)OCR/ECAR ratio, B) Non- mitochondrial respiration, C) Basal respiration, D) Proton leak, E) ATP-production, F) Spare respiratory capacity, G) Maximal respiration, H) non-glicolitic acidification, I) Glycolysis, J) Glicolytic capacity, K) Glycolytic reserve. Two independent experiments were conducted, each with five replicates, using two different cell lots. Statistical analysis was performed using the 2-way ANOVA test with Dunn’s post-test. ***<0,0001 **p < 0.01, *p < 0.05.
(TIF)

S2 Fig Growth curve of *L*. *amazonensis* and *L*. *braziliensis* parasites over 4 days. The parasites were treated with metabolic inhibitors for 1 hour and cultured for 4 days to determine the growth curve. The experiments were performed in triplicate.A) Growth curve of *L*. *amazonensis* B) Growth curve of *L*. *braziliensis*. Statistical analysis was conducted using the 2-way ANOVA test with Dunn’s post-test. *p < 0.05.
(TIF)

S3 Fig Parasite viability 5 days after the end of the infection. To assess parasite viability, BMDMs were treated with inhibitors for 1 hour either before or after infection, and the infection lasted 24 hours with *L*. *amazonensis* or *L*. *braziliensis*. After the infection period, the RPMI medium was replaced with Schneider’s medium, and cultivation continued for an additional 5 days to count the viable parasites. 2DG, 2—Deoxyglucose; OLG, Oligomycin; Ant A, Antimycin; DMSO, Dimethyl sulfoxide. The experiments were performed in triplicate. A) BMDMs were treated with inhibitors for 1 hour before the infection B) BMDMs were treated with inhibitors for 1 hour after infection. Statistical analysis was conducted using the 2-way ANOVA test with Dunn’s post-test. *p < 0.05.
(TIF)

S4 Fig Cellular viability after treatment with metabolic inhibitors. The cultures were treated with different concentrations of each metabolic inhibitor for 1 hour, washed, stained with AlamarBlue (a viability indicator dye), incubated for 72 hours, and subjected to spectrophotometry. For our dead control, we cultivated the cells in sterile water for injection. 2DG, 2—Deoxyglucose; PFK15, Phosphofructokinase; C75; ETMX, Etomoxir; OLG, Oligomycin; Ant A, Antimycin; ROT, Rotenone; FCCP, Carbonyl cyanide-p-trifluoromethoxyphenylhydrazone; MDVI, 3-(2,4-Dichloro-5-methoxyphenyl) -2,3-dihydro-2-thioxo-4(1H)-quinazolinone3-(2,4-Dichloro-5-methoxyphenyl)-2-sulfanyl-4(3H)-quinazolinone; DMSO, Dimethyl sulfoxide. The experiments were performed in triplicate. Statistical analysis was conducted using the Kruskal-Wallis test and Dunn’s post-test.
(TIF)

S5 Fig Images of macrophages at binding and phagocytosis. The BMDMs were treated with metabolic inhibitors for 1 hour before infection and then infected for 30 minutes for binding and 2 hours for phagocytosis. A) *La*, *L*. *amazonensis*; 2DG, 2-deoxyglucose; OLG, Oligomycin; Ant, Antimycin B) *Lb*, *L*.*braziliensis*; 2DG, 2-deoxyglucose; OLG, Oligomycin; Ant, Antimycin.
(TIF)

S1 Table Concentration of Metabolic Inhibitors utilized in macrophage infection with *Leishmania spp*. (TIF)

S1 Data FIG1A- FIG1N. This excel table outlines the experimental setup for evaluating metabolic parameters in macrophages (MO) and those infected with *Leishmania* species (*Leishmania amazonensis* [LA] and *Leishmania braziliensis* [LB]). These results concern Fig 1A to 1N –**Assessment of metabolic alterations in Leishmania-infected bone marrow-derived macrophages. FIG2A-FIG2B**. MitoTracker Green columns A-C includes three groups: (A) Control group (Mock),(B) Leishmania amazonensis (La) infection,(C) Leishmania braziliensis (Lb) infection**. MitoTracker Red** columns E-G) shows the same groups: (E) Control group (Mock) (F) Leishmania amazonensis (La) infection (G) Leishmania braziliensis (Lb) infection. Five replicates were used for each experimental condition. These results concern Fig2A and Fig2B- **Fig 2. *Leishmania* spp promote increased mitochondrial mass and membrane potential. FIG3A- FIG3B** the results concern the infection rate and parasite load of macrophages infected with *Leishmania amazonensis* and *Leishmania braziliensis*. **Evaluation of infection rate and parasite load in macrophages treated with metabolic inhibitors during *L*. *amazonensis* or *L*. *braziliensis* infections**. **Fig 4. Infection Index:** concern the results shown in Fig 4 Infection index is calculated by multiplying the infection rate by the number of amastigotes. The values represent the average of parasite load and infection rate data.—**Determination of infection index following metabolic inhibitor treatment. FIG 5**. Results associated to the Fig 5 **Inhibitor metabolic pathways did not affect binding and phagocytosis of the parasites** Binding and phagocytosis in macrophages infected with *Leishmania amazonensis* and *Leishmania braziliensis*: **FIG6B-Fig6F** Results associated to the graphs shown in Fig 6 that evaluates mitochondrial reactive oxygen species (ROS) production: **Effects of 2DG and Antimycin A treatment on ROS levels and *Leishmania* parasite load in infected macrophages.**
(XLSX)

## References

[1] Gordon S, Martinez-Pomares L. Physiological roles of macrophages. 1945; 10.1007/s00424-017-1945-7 PMC5362657 28185068

[2] Van den Bossche J, O’Neill LA, Menon D. Macrophage Immunometabolism: Where Are We (Going)? Trends Immunol. 2017 Jun 1;38(6):395–406. doi: 10.1016/j.it.2017.03.001 28396078

[3] Russell DG, Huang L, VanderVen BC. Immunometabolism at the interface between macrophages and pathogens. Nat Rev Immunol 2019 195 [Internet]. 2019 Jan 24 [cited 2024 Oct 7];19(5):291–304. Available from: https://www.nature.com/articles/s41577-019-0124-9. doi: 10.1038/s41577-019-0124-9 30679807 PMC7032560

[4] O’Neill LAJ, Kishton RJ, Rathmell J. A guide to immunometabolism for immunologists. Nat Rev Immunol. 2016;16(9):553–65. doi: 10.1038/nri.2016.70 27396447 PMC5001910

[5] Czyz DM, Willett JW, Crosson S. Brucella abortus Induces a Warburg Shift in Host Metabolism That Is Linked to Enhanced Intracellular Survival of the Pathogen. J Bacteriol [Internet]. 2017 Aug 1 [cited 2024 Oct 7];199(15). Available from: https://pubmed.ncbi.nlm.nih.gov/28559292/. doi: 10.1128/JB.00227-17 28559292 PMC5512224

[6] Codo AC, Davanzo GG, Monteiro L de B, de Souza GF, Muraro SP, Virgilio-da-Silva JV, et al. Elevated Glucose Levels Favor SARS-CoV-2 Infection and Monocyte Response through a HIF-1α/Glycolysis-Dependent Axis. Cell Metab. 2020;32(3):437–446.e5. 32697943 10.1016/j.cmet.2020.07.007 PMC7367032

[7] Umar S, Palasiewicz K, Meyer A, Kumar P, Prabhakar BS, Volin M V., et al. Inhibition of IRAK4 dysregulates SARS-CoV-2 spike protein-induced macrophage inflammatory and glycolytic reprogramming. Cell Mol Life Sci [Internet]. 2022 Jun 1 [cited 2024 Oct 7];79(6). Available from: https://pubmed.ncbi.nlm.nih.gov/35588018/. doi: 10.1007/s00018-022-04329-8 35588018 PMC9118817

[8] Janžič L, Repas J, Pavlin M, Zemljić-Jokhadar Š, Ihan A, Kopitar AN. Macrophage polarization during Streptococcus agalactiae infection is isolate specific. Front Microbiol [Internet]. 2023 [cited 2024 Oct 7];14. Available from: https://pubmed.ncbi.nlm.nih.gov/37213504/. doi: 10.3389/fmicb.2023.1186087 37213504 PMC10192866

[9] Alvar J, Vélez ID, Bern C, Herrero M, Desjeux P, Cano J, et al. Leishmaniasis worldwide and global estimates of its incidence. PLoS One. 2012;7(5). doi: 10.1371/journal.pone.0035671 22693548 PMC3365071

[10] Desjeux P. Leishmaniasis: current situation and new perspectives. Comp Immunol Microbiol Infect Dis. 2004;27(5):305–18. doi: 10.1016/j.cimid.2004.03.004 15225981

[11] De Oliveira CI, Brodskyn CI. The immunobiology of Leishmania braziliensis infection. Front Immunol. 2012;3(JUN):1–9. doi: 10.3389/fimmu.2012.00145 22701117 PMC3370302

[12] Amato VS, Tuon FF, Bacha HA, Neto VA, Nicodemo AC. Mucosal leishmaniasis. Current scenario and prospects for treatment. Vol. 105, Acta Tropica. 2008. p. 1–9. doi: 10.1016/j.actatropica.2007.08.003 17884002

[13] Ortiz D, Forquer I, Boitz J, Soysa R, Elya C, Fulwiler A, et al. Targeting the cytochrome bc1 complex of Leishmania parasites for discovery of novel drugs. Antimicrob Agents Chemother. 2016;60(8):4972–82. doi: 10.1128/AAC.00850-16 27297476 PMC4958202

[14] Carneiro PP, Conceição J, Macedo M, Magalhães V, Carvalho EM, Bacellar O. The role of nitric oxide and reactive oxygen species in the killing of Leishmania braziliensis by monocytes from patients with cutaneous leishmaniasis. PLoS One. 2016;11(2):1–16. doi: 10.1371/journal.pone.0148084 26840253 PMC4739692

[15] Caradonna KL, Engel JC, Jacobi D, Lee CH, Burleigh BA. Host metabolism regulates intracellular growth of trypanosoma cruzi. Cell Host Microbe [Internet]. 2013;13(1):108–17. Available from: doi: 10.1016/j.chom.2012.11.011 23332160 PMC3560928

[16] Eisenreich W, Rudel T, Heesemann J, Goebel W. To Eat and to Be Eaten: Mutual metabolic adaptations of immune cells and intracellular bacterial pathogens upon infection. Front Cell Infect Microbiol. 2017;7(JUL):1–26. doi: 10.3389/fcimb.2017.00316 28752080 PMC5508010

[17] McConville MJ, de Souza D, Saunders E, Likic VA, Naderer T. Living in a phagolysosome; metabolism of Leishmania amastigotes. Trends Parasitol. 2007;23(8):368–75. doi: 10.1016/j.pt.2007.06.009 17606406

[18] Naderer T, McConville MJ. The Leishmania-macrophage interaction: A metabolic perspective. Cell Microbiol. 2008;10(2):301–8. doi: 10.1111/j.1462-5822.2007.01096.x 18070117

[19] Opperdoes FR, Coombs GH. Metabolism of Leishmania: proven and predicted. Trends Parasitol. 2007;23(4):149–58. doi: 10.1016/j.pt.2007.02.004 17320480

[20] Moreira D, Rodrigues V, Abengozar M, Rivas L, Rial E, Laforge M, et al. Leishmania infantum Modulates Host Macrophage Mitochondrial Metabolism by Hijacking the SIRT1-AMPK Axis. PLoS Pathog. 2015;11(3):1–24. doi: 10.1371/journal.ppat.1004684 25738568 PMC4349736

[21] Ospina HA, Guay-Vincent MM, Descoteaux A. Macrophage Mitochondrial Biogenesis and Metabolic Reprogramming Induced by Leishmania donovani Require Lipophosphoglycan and Type I Interferon Signaling. MBio [Internet]. 2022 Dec 1 [cited 2024 Oct 7];13(6). Available from: https://journals.asm.org/doi/10.1128/mbio.02578-22. 10.1128/mbio.02578-22 PMC9764995 36222510

[22] Ty MC, Loke P, Alberola J, Rodriguez-Cortes A, Rodriguez-Cortes A. Immuno-metabolic profile of human macrophages after Leishmania and Trypanosoma cruzi infection. PLoS One. 2019;14(12):1–12. doi: 10.1371/journal.pone.0225588 31841511 PMC6913957

[23] Dou L, Chen YF, Cowan PJ, Chen XP. Extracellular ATP signaling and clinical relevance. Clin Immunol [Internet]. 2018;188:67–73. Available from: doi: 10.1016/j.clim.2017.12.006 29274390

[24] Bhattacharyya YC and SN. Leishmania donovani restricts mitochondrial dynamics to enhance miRNP stability and target RNA repression in host macrophages Running. 2017;(Ld). 10.1091/mbc.E16-06-0388 PMC5509422 28539410

[25] Ohms M, Ferreira C, Busch H, Wohlers I, Guerra de Souza AC, Silvestre R, et al. Enhanced Glycolysis Is Required for Antileishmanial Functions of Neutrophils Upon Infection With Leishmania donovani. Front Immunol [Internet]. 2021 Mar 19 [cited 2024 Oct 7];12:632512. Available from: www.frontiersin.org. doi: 10.3389/fimmu.2021.632512 33815385 PMC8017142

[26] Remmerie A, Scott CL. Macrophages and lipid metabolism. Cell Immunol [Internet]. 2018;330(October 2017):27–42. Available from: doi: 10.1016/j.cellimm.2018.01.020 29429624 PMC6108423

[27] Rabhi S, Rabhi I, Trentin B, Piquemal D, Regnault B, Goyard S, et al. Lipid droplet formation, their localization and dynamics during leishmania major macrophage infection. PLoS One. 2016;11(2):1–19. doi: 10.1371/journal.pone.0148640 26871576 PMC4752496

[28] Chandel NS. Mitochondrial complex III: An essential component of universal oxygen sensing machinery? Respir Physiol Neurobiol. 2010 Dec 31;174(3):175–81. doi: 10.1016/j.resp.2010.08.004 20708106 PMC2991558

[29] Smilkstein MJ, Forquer I, Kanazawa A, Kelly JX, Winter RW, Hinrichs DJ, et al. A drug-selected Plasmodium falciparum lacking the need for conventional electron transport. Mol Biochem Parasitol. 2008 May 1;159(1):64–8. doi: 10.1016/j.molbiopara.2008.01.002 18308406 PMC2396451

