# Supplementary figures and images for: Pharmacological inhibition of key metabolic pathways attenuates Leishmania spp infection in macrophages
Source: PLoS Negl Trop Dis. 2025 Jan 7;19(1):e0012763. doi: 10.1371/journal.pntd.0012763 (PMC11756801; doi:10.1371/journal.pntd.0012763)

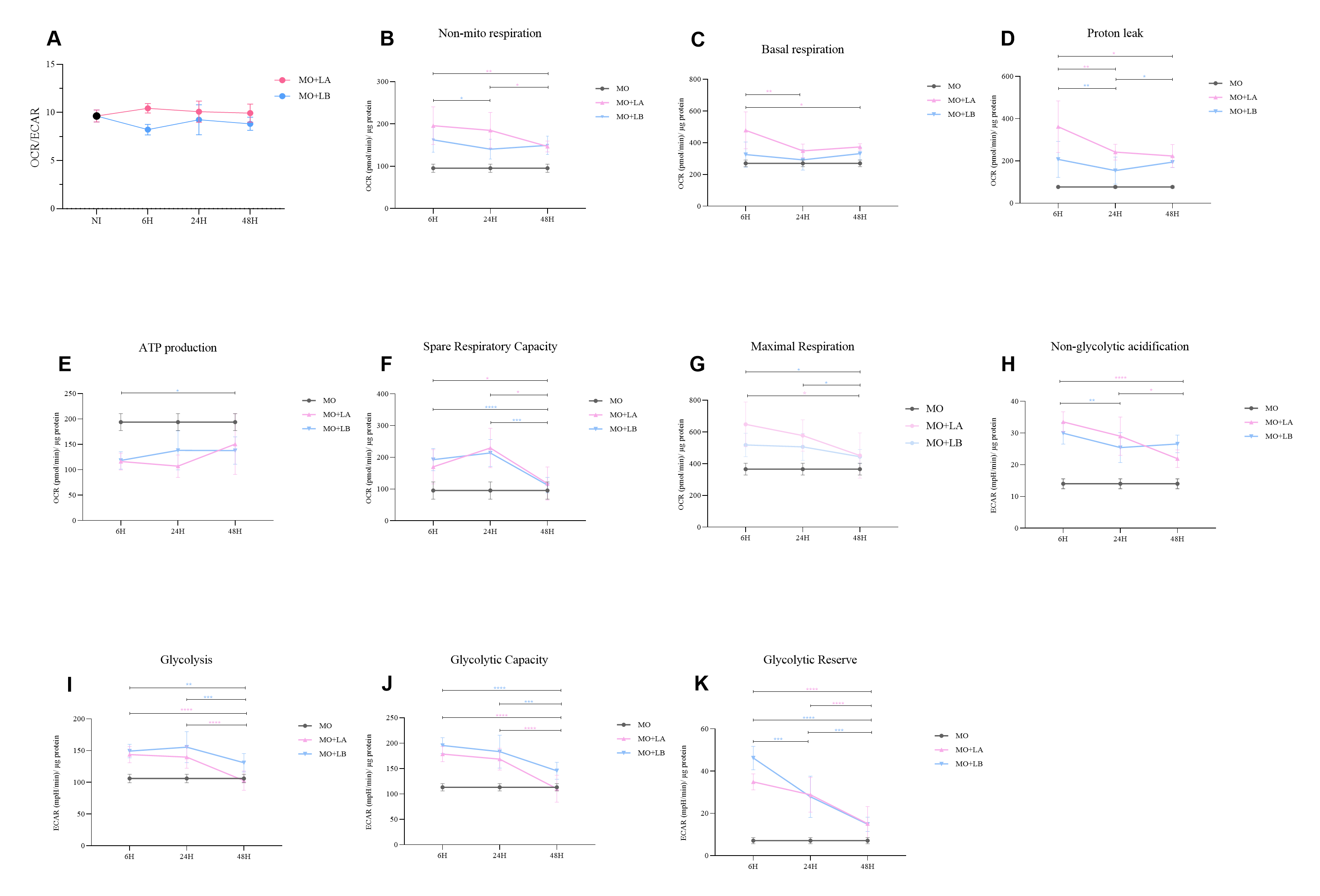

Supplement: S1 Fig — Real-time extracellular acidification rate (ECAR) analysis of bone marrow-derived macrophages from uninfected (mock) C57BL/6 mice and those infected with La (Leishmania amazonensis) or Lb (Leishmania braziliensis) for 6 hours, followed by incubation for an additional 18 or 42 hours post-infection. Real-time oxygen consumption rate (OCR) analysis was also performed under the same conditions. This data highlights metabolic differences in macrophages at distinct infection time points. A)OCR/ECAR ratio, B) Non- mitochondrial respiration, C) Basal respiration, D) Proton leak, E) ATP-production, F) Spare respiratory capacity, G) Maximal respiration, H) non-glicolitic acidification, I) Glycolysis, J) Glicolytic capacity, K) Glycolytic reserve. Two independent experiments were conducted, each with five replicates, using two different cell lots. Statistical analysis was performed using the 2-way ANOVA test with Dunn’s post-test. ***<0,0001 **p < 0.01, *p < 0.05. (TIF) [file pntd.0012763.s001.tif]

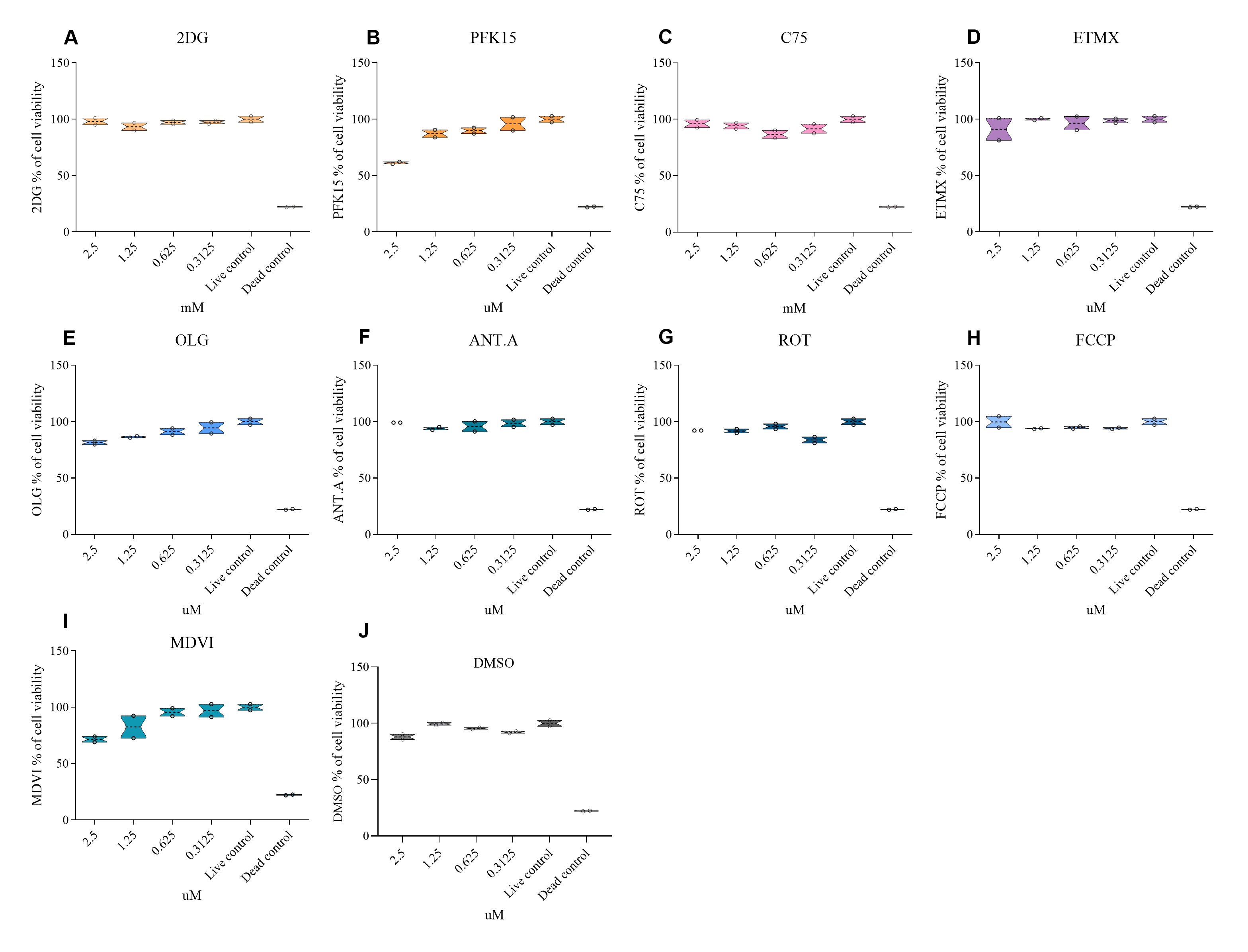

Supplement: S4 Fig — The cultures were treated with different concentrations of each metabolic inhibitor for 1 hour, washed, stained with AlamarBlue (a viability indicator dye), incubated for 72 hours, and subjected to spectrophotometry. For our dead control, we cultivated the cells in sterile water for injection. 2DG, 2—Deoxyglucose; PFK15, Phosphofructokinase; C75; ETMX, Etomoxir; OLG, Oligomycin; Ant A, Antimycin; ROT, Rotenone; FCCP, Carbonyl cyanide-p-trifluoromethoxyphenylhydrazone; MDVI, 3-(2,4-Dichloro-5-methoxyphenyl) -2,3-dihydro-2-thioxo-4(1H)-quinazolinone3-(2,4-Dichloro-5-methoxyphenyl)-2-sulfanyl-4(3H)-quinazolinone; DMSO, Dimethyl sulfoxide. The experiments were performed in triplicate. Statistical analysis was conducted using the Kruskal-Wallis test and Dunn’s post-test. (TIF) [file pntd.0012763.s004.tif]

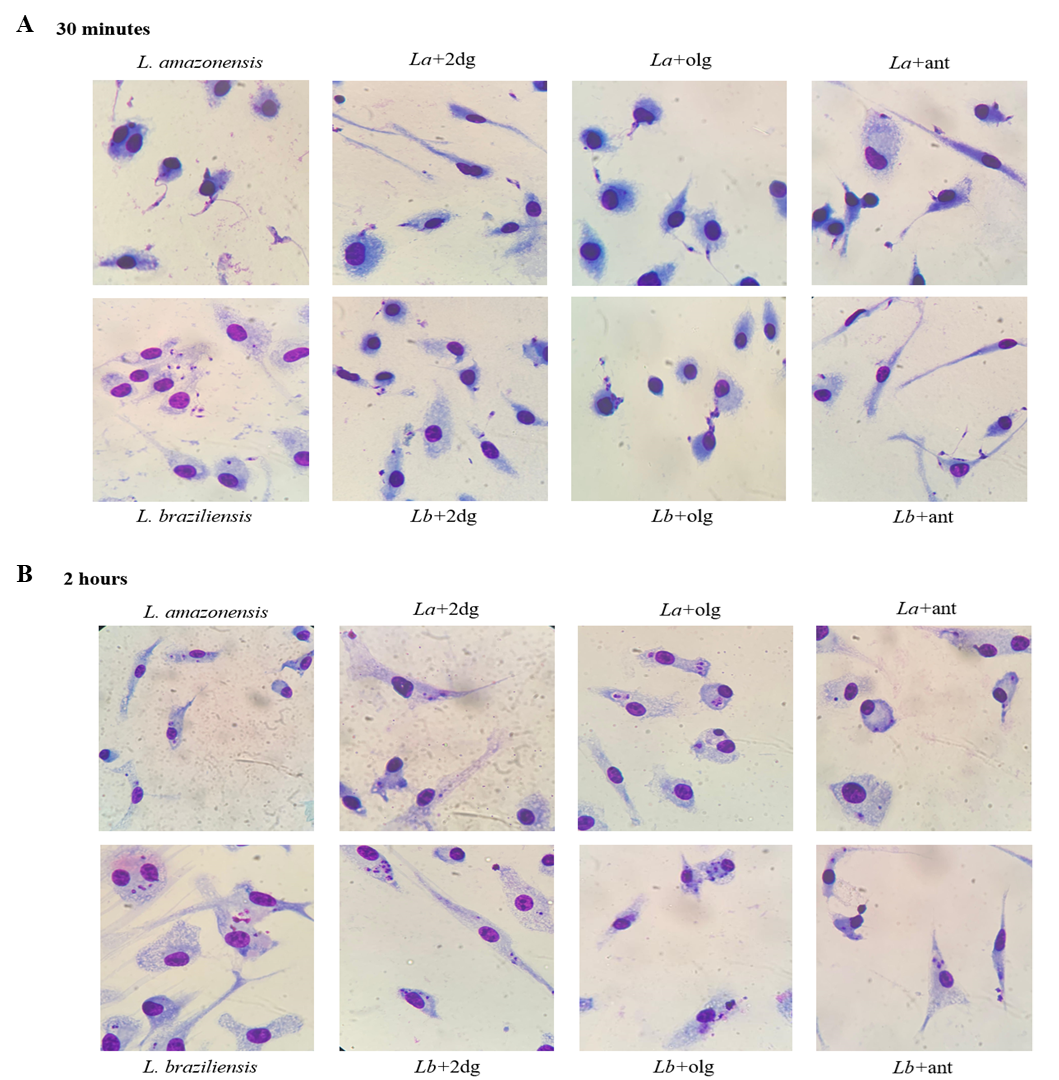

Supplement: S5 Fig — The BMDMs were treated with metabolic inhibitors for 1 hour before infection and then infected for 30 minutes for binding and 2 hours for phagocytosis. A) La, L. amazonensis; 2DG, 2-deoxyglucose; OLG, Oligomycin; Ant, Antimycin B) Lb, L.braziliensis; 2DG, 2-deoxyglucose; OLG, Oligomycin; Ant, Antimycin. (TIF) [file pntd.0012763.s005.tif]

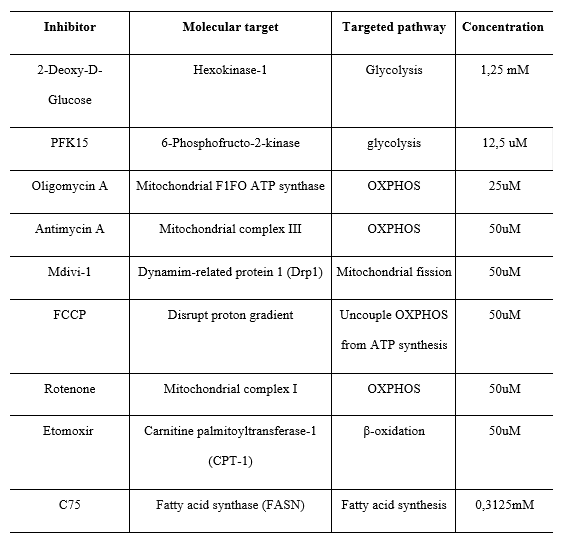

Supplement: S1 Table — (TIF) [file pntd.0012763.s006.tif]
